# Characterization of natural bactericidal antibody against *Haemophilus influenzae* type a in Canadian First Nations: A Canadian Immunization Research Network (CIRN) Clinical Trials Network (CTN) study

**DOI:** 10.1371/journal.pone.0201282

**Published:** 2018-08-15

**Authors:** Eli B. Nix, Joshua Choi, Christina Anthes, Gabrielle N. Gaultier, Joelle Thorgrimson, Andrew D. Cox, Raymond S. W. Tsang, William G. McCready, Douglas Boreham, Marina Ulanova

**Affiliations:** 1 Northern Ontario School of Medicine, Thunder Bay, Ontario, Canada; 2 Department of Biology, Lakehead University, Thunder Bay, Ontario, Canada; 3 National Research Council, Ottawa, Canada; 4 National Microbiology Laboratory, Public Health Agency of Canada, Winnipeg, Manitoba, Canada; University Medical Center Utrecht, NETHERLANDS

## Abstract

During the last two decades, *Haemophilus influenzae* serotype a (Hia) emerged as an important cause of invasive disease in Canadian First Nations and Inuit, and Alaskan Native populations, with the highest rates reported in young children. Immunocompetent adults, in contrast to children, do not typically develop invasive Hia disease. To clarify factors responsible for an increased burden of invasive Hia disease in certain population groups we studied serum bactericidal activity (SBA) against Hia and quantified IgG and IgM specific to Hia capsular polysaccharide in healthy adult members of two First Nations communities: 1) with reported cases of invasive Hia disease (Northern Ontario, NO), and 2) without reported cases (Southern Ontario, SO), in comparison to non-First Nations living in proximity to the NO First Nations community, and non-First Nations elderly non-frail Canadians from across the country (total of 110 First Nations and 76 non-First Nations). To elucidate the specificity of bactericidal antibodies, sera were absorbed with various Hia antigens. Naturally acquired SBA against Hia was detected at higher rates in First Nations (NO, 80%; SO, 96%) than non-First Nations elderly Canadians (64%); the SBA titres in First Nations were higher than in non-First Nations elderly Canadians (P<0.001) and NO non-First Nations adults (P>0.05). Among First Nations, SBA was mediated predominantly by IgM, and by both antibodies specific to Hia capsular polysaccharide and lipooligosaccharide. Conclusions: The SBA against Hia is frequently present in sera of First Nations adults regardless of the burden of Hia disease observed in their community; it may represent part of the natural antibody repertoire, which is potentially formed in this population under the influence of certain epigenetic factors. Although the nature of these antibodies deserves further studies to understand their origin, the data suggest that they may represent important protective mechanism against invasive Hia disease.

## Introduction

*Haemophilus influenzae* is a Gram-negative bacterial pathogen, which colonizes human respiratory and genital tracts and can cause a wide range of local and systemic infections including otitis media, sinusitis, pneumonia, epiglottitis, meningitis, and septic arthritis. Many *H*. *influenzae* strains have a polysaccharide capsule, which protects bacteria against host responses and acts as the major antigen. On the basis of the antigenic properties of capsular polysaccharides, 6 serological types are distinguished (a-f) while non-encapsulated *H*. *influenzae* are referred to as non-typeable (NTHi) [1].

*H*. *influenzae* type b (Hib) was the major cause of pediatric bacterial meningitis prior to the introduction of conjugate Hib vaccine in late 1980s-early 1990s [2]. Since publicly-funded immunization against Hib had been implemented, a great decline in the incidence rates of invasive Hib disease occurred worldwide; most cases of invasive *H*. *influenzae* disease are now caused by NTHi, which primarily affects newborns and immunocompromised individuals [3].

However, in certain geographic areas and populations, *H*. *influenzae* type a (Hia) has been reported as a significant cause of severe invasive infections mainly affecting young children, and similar in presentation to Hib disease in the pre-Hib vaccine era. Remarkably, most of the reported cases of invasive Hia disease occur among North American Indigenous populations of Alaska, Northern Canada, and US Southwest as well as in Aboriginal people of Australia [4–9]. In some areas and populations, the incidence rates of invasive Hia disease are now close to those reported for invasive Hib disease prior to Hib vaccine introduction [9].

Molecular-genetic characteristics of clinical Hia isolates have been studied, and it appears that certain strain-specific features are associated with their enhanced virulence and may be responsible for the most severe forms of infection [10, 11]. However, these studies did not answer the question why the highest incidence rates of invasive Hia disease are found among North American Indigenous peoples.

As susceptibility to infections depends on dynamic interactions between pathogen virulence characteristics and host defenses, we explored a possibility that certain populations of Indigenous peoples may have a decreased capacity of building protective immunity against Hia. In our recent study, we analyzed naturally acquired serum bactericidal antibody specific to Hia in Canadian Indigenous and non-Indigenous adult individuals residing in the same geographic area where increased incidence rates of invasive Hia disease were reported, i.e. Northwestern Ontario [12, 13]. To our surprise, both healthy and immunocompromised Indigenous adults expressed significantly higher titers of serum bactericidal antibody against Hia compared to their non-Indigenous counterparts; moreover, the bactericidal activity was mainly attributed to IgM antibodies suggesting a recent exposure to the pathogen although these antibodies can also be natural IgM produced by B1b cells [14, 15].

In this study, we extended our observations to other Canadian Indigenous and non-Indigenous populations. In addition, to get insight into the origin of natural Hia antibodies we addressed the following questions: 1) Is the prevalence of Hia bactericidal antibodies dependent of the burden of the invasive disease in a community? 2) Are bactericidal Hia antibodies strain specific? 3) Which antigenic components of Hia are recognized by these antibodies?

## Materials and methods

### Ethics statement

In designing and conducting this study, we adhered to the principles of Ownership, Control, Access, and Possession (OCAP) as defined by the National Aboriginal Health Organization [16], and the guidelines of Canadian Tri-Council Policy Statement: Ethical Conduct for Research Involving Humans (TCPS2), specifically those outlined in Chapter 9: Research Involving First Nations, Inuit and Métis Peoples of Canada [17]. The study was approved by the Lakehead University Research Ethics Board (Thunder Bay, Ontario).

### Study participants

Adult members of two Ojibwa First Nations communities located in different parts of the province of Ontario were recruited. One from Northwestern Ontario (NWFN) and the other from Southern Ontario (SFN). In addition, we recruited Non-First Nations residents of Kenora (NFNK), which is a small city in Northwestern Ontario with population of 15,000. The age of study participants was between 18 and 89 years, the average female/male ratio was 1.2. The demographics of these groups are presented in Table 1. Serum samples were obtained under informed written consent from adult volunteers who self-declared generally healthy, during January-November 2015. After clotting, the samples were centrifuged at 4°C and put into the -80°C freezer. Sera were stored at -80°C prior to analysis.

**Table 1:** Demographics of study participants.

Group	Number	Age Mean (Median)	Age Range	Age ≥65 (%)	% Female
Northwestern Ontario FN	60	38.4 (36.5)	18–68	2 (3.3)	55
Southern Ontario FN	50	48.9 (52)	20–80	5 (10)	52
Non-FN (Kenora)	26	45.8 (53)	20–89	4 (15.4)	53.8
Non-FN Senior Canadians [18]	50	73.8	≥65	50 (100)	N/A

FN, First Nations.

For comparison, we included non-First Nations elderly non-frail Canadians (NFNC) from across the country (≥65 years, mean age 73.8) collected in September-October 2011 by PHAC/CIHR Influenza Research Network (the population is described in [18]). Fifty serum samples from this cohort (anonymized as was permitted by the original study participants) were kindly provided by Dr. Brian Ward.

### Bacterial strains and culture conditions

The characteristics of *H*. *influenzae* type a clinical isolates are summarized in Table 2 and described previously [19]. All *H*. *influenzae* were grown on brain heart infusion (BHI) agar plates supplemented with 10 μg/ml hemin and 1 μg/ml nicotine adenine dinucleotide (NAD) (sBHI) at 37°C with 5% CO_2_.

**Table 2:** Characteristics of *Haemophilus influenzae* type a clinical isolates.

Isolate ID	Biotype	Sequence type	Invasive isolate	Province	Patient age	Site of isolation
08–191	II	23*	Yes	Ontario	47 years	Blood
13–155	I	62^+^	Yes	Manitoba	60 years	CSF
11–139	II	23*	Yes	Manitoba	28 years	Blood
13–240	I	4*	Yes	Manitoba	1 year	Blood
14–61	II	23*	No	Ontario	6 months	Ear
11–173	II	1035*	Yes	Manitoba	1 year	Blood
04–001	II	405*	Yes	Manitoba	2 months	Blood

CSF, cerebrospinal fluid

*Clonal division I: ST-23, ST-405, and ST-1035 belong to the same clonal complex while ST-4 is genetically different and shares no multilocus sequence typing gene alleles with ST-23.

^**+**^Clonal division II.

### Antibody analysis

Concentrations of serum IgG and IgM antibodies specific to Hia capsular polysaccharide (PS) were measured in study participants and intravenous IgG (IVIG) prepared from a pool of over a thousand Canadian donors (Privigen, CSL Behring, Ottawa, Canada) using ELISA as described by [14]. The lower limit of detection was 0.10 μg/ml for IgG and 0.01 μg/ml for IgM antibody.

### Serum bactericidal assay (with exogenous complement)

Serum bactericidal activity in presence of exogenous (baby rabbit) complement was tested as described by [14], using Hia isolate 08–191. The results (SBA titres) were reported as the reciprocal serum dilution required to kill ≥50% of the initial bacterial inoculum [20]. Discontinuous titres below the lower detection limit of 16 were reported as 8 for statistical purposes (14).

### Bacterial killing assay (with endogenous complement)

*H*. *influenzae* type a isolates were grown overnight at 37°C, 5% CO_2_, harvested at log phase, diluted in SBA buffer consisting of Hanks' buffered salt solution supplemented with 10 μg/ml hemin and 1 μg/ml NAD. Bacteria were mixed with serum (S) or heat inactivated serum (HIS) (30 minutes in water bath at 56°C) and incubated for one hour at 37°C, 5% CO_2_. Next bacteria were drop plated on sBHI agar plates and incubated for 18 hours at 32°C followed by counting colony-forming units (CFU). Results were reported as percent killing: [(CFU_HIS_−CFU_S_)/CFU_HIS_] x 100.

For absorption of antigen-specific antibody, serum was mixed with an equal volume of an antigen of interest (Table 3) in a final concentration of 100 μg/ml, and incubated on a rotator at 4°C overnight. Mock absorbed serum was mixed with an equal volume of PBS. Following incubation, the samples were centrifuged at 9,000 x *g* for 10 minutes, and the supernatants were used for testing.

**Table 3:** Antigens used to absorb pooled human serum.

Antigen	Source
Hia polysaccharide from isolate 11–139	NRC prepared
Hib polysaccharide	NRC prepared
Hia lipooligosaccharide from isolate 11–139	NRC prepared
*Streptococcus pneumoniae* polysaccharide 6B (ATCC 228-X)	Cederlane Labs, Burlington, ON
*H*. *influenzae* Protein D	NRC prepared
Human serum albumin	Sigma

Hia, *H*. *influenzae* type a; Hib, *H*. *influenzae* type b; NRC, National Research Council of Canada; ATCC, American Type Culture Collection

The bacterial killing assay was also performed in the presence of 10 mM Mg^2+^ EGTA, which blocks the classical as well as the lectin pathway of complement activation.

### Complement activity

The CH50 Eq immunoassay (Cedarlane, Burlington, Canada) was used according to the manufacturer’s protocol; results were expressed as CH50 equivalent units per ml.

### Statistical analysis

For analysis, each set of data represented at least 2 independent experiments, and each independent experiment was done in triplicate. We performed transformation of log_10_ data for exogenous serum bactericidal assay prior to analysis. Statistical significance was assessed using Graph-Pad Prism 5 (GraphPad Software Inc., San Diego, CA, USA.) The various statistical tests used are specified in the figure legends.

## Results

### Natural antibodies against Hia are present at higher levels in First Nations than non-First Nations individuals regardless of their potential exposure to the pathogen

To compare natural immunity against Hia in populations with different exposure to the pathogen, we studied the serum bactericidal activity (SBA, with baby rabbit complement) in residents of one Northwestern Ontario First Nation (NWFN, n = 60), one First Nation of Southern Ontario (SFN, n = 50), as well as non-First Nations (n = 26) residents of Kenora, Northwestern Ontario (NFNK). Increased incidence rates of invasive Hia disease for all ages have been reported from Northwestern, but not from Southern Ontario (in comparison to the data of the whole province of Ontario) [21]. Concentrations of serum IgG and IgM specific to Hia capsular polysaccharide antigen (PS) were determined in 60 NWFN, 28 SFN, and 10 NFNK participants. In addition, serum bactericidal activity against Hia (n = 50) and the antibody concentrations (n = 17) were analyzed in 50 non-First Nations elderly non-frail Canadians from across the country, NFNC (≥65 years, mean age 73.8) [18].

As shown in Table 4, the highest titres of bactericidal Hia antibody were detected in the group of SFN. Both NWFN and SFN adults exhibited significantly higher SBA titres than NFNC (P<0.001); in all First Nations participants, there was a tendency to higher SBA titres than in NFNK (P>0.05). The bactericidal activity was below the lower detection limit in 20% of NWFN, 8% of SFN, 19.2% of NFNK, and 36% of NFNC. The detection rates were significantly lower in NFNC than in any other group (Table 4).

**Table 4:** Concentrations of IgM and IgG antibody specific for Hia polysaccharide and titres of bactericidal antibody against Hia 08–191 (with exogenous complement) in adult First Nations and non-First Nations individuals.

Study participants	SBA: GMT (95% CI); Detection rate (%)	IgM: GMC (95% CI) μg/ml	IgG: GMC (95% CI) μg/ml	IgM/IgG ratio
(1) Northwestern FN	506.1 (278.2–920.8); 48 (80%)	2.09 (1.74–2.50)	1.47 (1.05–2.05)	1.42
(2) Southern FN	621.7 (375.6–1029); 46 (92%)	3.03 (2.04–4.51)	1.43 (0.84–2.42)	2.12
(3) Non-FN (Kenora)	285.1 (123–661); 21 (80.82%)	1.14 (0.74–1.75)	1.79 (0.90–3.54)	0.67
(4) Non-FN (Senior Canadians)	84.45 (46.4–153.7); 32 (64%)	1.10 (0.63–1.93)	1.40 (0.36–5.40)	0.79

CI, confidence intervals; FN, First Nations; GMC, geometrical mean concentration; GMT, geometrical mean titre; Hia, *H*. *influenzae* type a; SBA, serum bactericidal activity

Statistical significance: for SBA titers, P<0.001 between groups (1) and (4) and (2) and (4). For IgM, P<0.01 between groups (1) and (2), (2) and (4), (2) and (3) (One-way ANOVA with Tukey's post hoc test). For IgG, no significant difference between groups. For SBA detection rates, P<0.001 between groups (1) and (4); P<0.0001 between groups (2) and (4); P<0.01 between groups (3) and (4) (Fisher’s exact test).

The highest IgM antibody geometrical mean concentrations were found in SFN, followed by NWFN, and the lowest were found in NFNC (P<0.01). The group of NFNK had significantly lower IgM antibody concentrations than SFN (P<0.01) as well as lower SBA titres (not statistically significant), while both populations live in the same region. The concentrations of anti-Hia PS IgG did not noticeably differ among the groups; however, in both groups of First Nations adults, concentrations of IgM prevailed over IgG, with a reverse IgM/IgG ratio in non-First Nations individuals (Table 4).

A weak negative correlation of SBA titres with age, and of Hia-specific IgM (but not IgG) concentrations with age was detected (S1–S3 Figs).

### Serum bactericidal activity against Hia in First Nations individuals extends beyond locally circulating Hia strain belonging to the sequence type (ST)-23

As shown on Table 4, adult First Nations individuals exhibit high titres of bactericidal antibody specific to the Hia strain 08–191 (was isolated in 2008 from the blood of a First Nation adult of Northwestern Ontario who developed invasive Hia disease), which belongs to the sequence type ST-23 currently circulating in the region [13, 22–24]. In the next series of experiments, we sought to test whether the naturally acquired bactericidal activity in this population extends beyond the locally circulating Hia strains and is directed towards invasive and non-invasive clinical Hia isolates of other sequence types from other areas.

**Fig 1:**
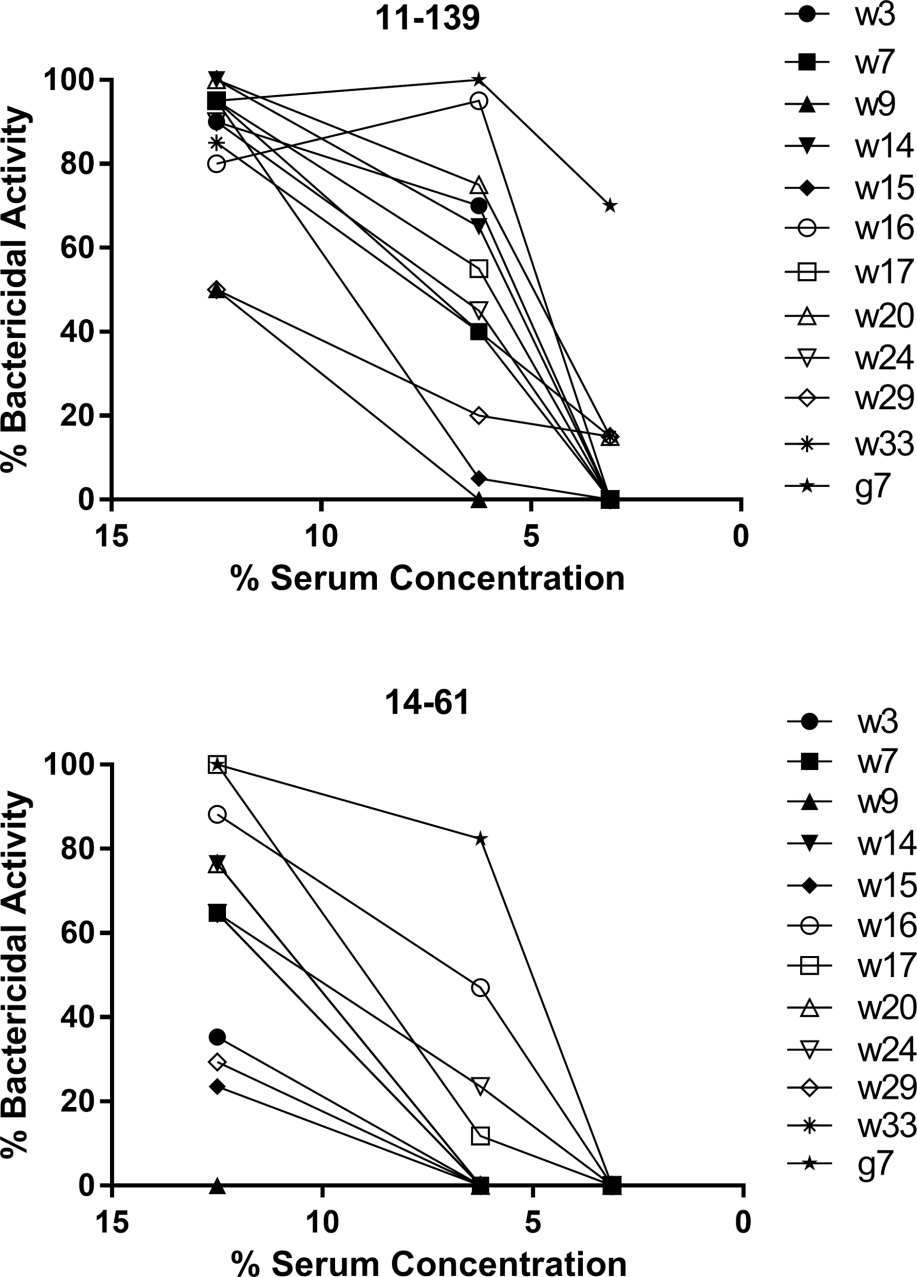
**Sensitivity of Hia isolates 11–139 (A) and 14–61 (B) to serum bactericidal effect varies among individual sera.** Bacteria were incubated for 1 hour at 37°C, 5% CO_2_ with 12 individual sera or heat inactivated sera at concentrations of 12.5%, 6.25% or 3.12% and then viable bacteria were enumerated. Bactericidal activity was determined by comparing the number of viable bacteria exposed to serum versus heat inactivated serum. Mean percentage bactericidal activity is indicated. The graphs are representative of at least two independent experiments, each performed in triplicate, which gave similar results.

**Fig 2:**
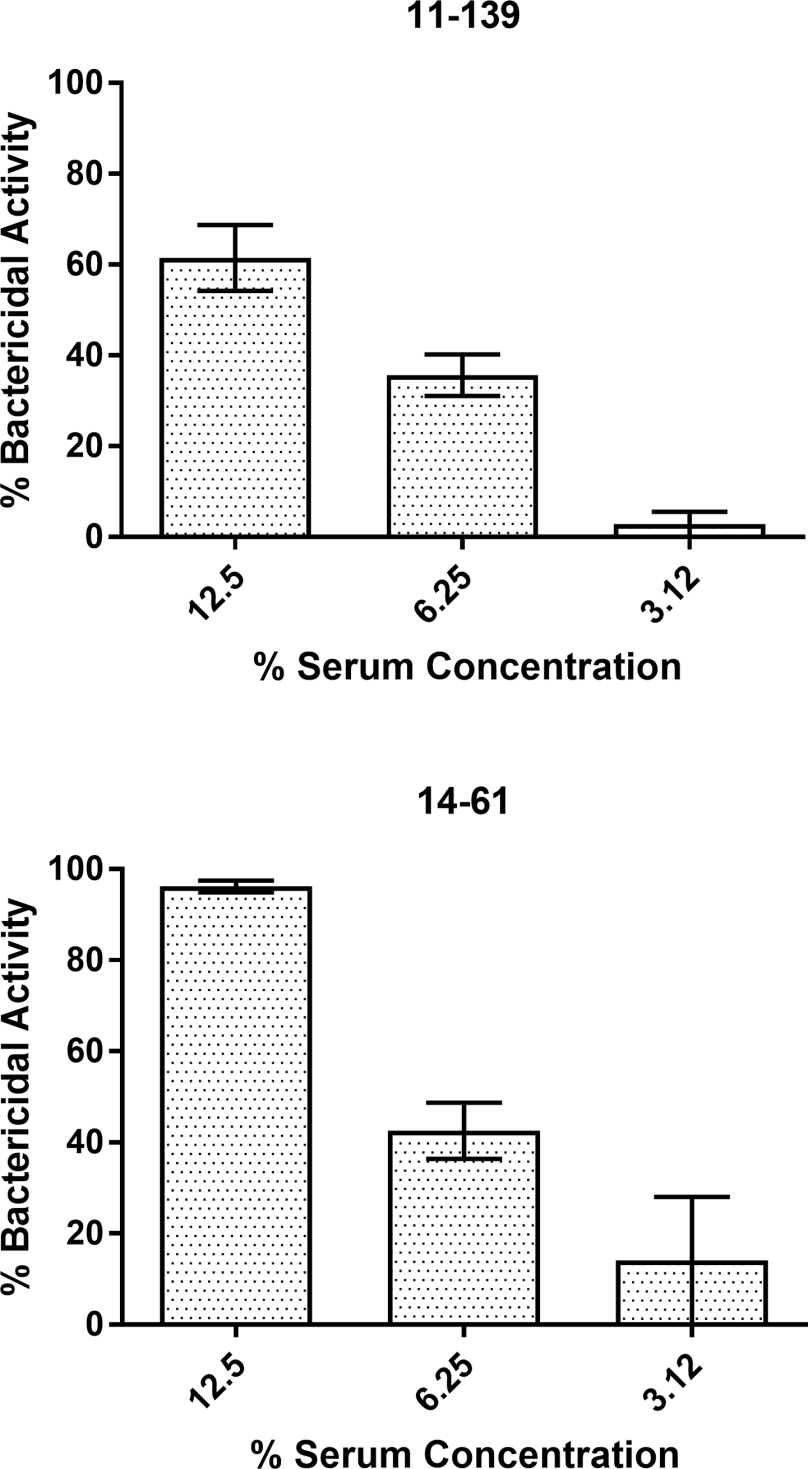
**Hia isolates 11–139 (A) and 14–61 (B) are sensitive to serum in a dose dependent manner**. Bacteria were incubated for 1 hour at 37°C, 5% CO_2_ with pooled serum or heat inactivated serum at concentrations of 12.5%, 6.25% or 3.12% and then viable bacteria were enumerated. Bactericidal activity was determined by comparing the number of viable bacteria exposed to serum versus heat inactivated serum. The graphs are representative of three independent experiments, each performed in triplicate, which gave similar results.

As bactericidal activity of antibody specific to encapsulated bacteria, such as *H*. *influenzae* or *Neisseria meningitidis*, may significantly depend on the source of complement [25], we optimized the serum bactericidal assay using endogenous rather than exogenous (baby rabbit) complement. Analysis of serum bactericidal activity of 12 randomly selected First Nations individuals showed that they exhibited the SBA geometrical mean titer of 608.9 (95% Confidence Intervals: 163.3–2271) when tested against Hia 08–191 using rabbit complement (S1 Table). Using endogenous complement in the serum bactericidal assay, we found a certain degree of variability among these sera in killing two other Hia isolates belonging to the same ST-23 as 08–191, i.e. 11–139 (invasive) and 14–61 (non-invasive) (Fig 1A and 1B). As we further tested the bactericidal activity of pooled serum of two individuals using endogenous complement (SBA titres using rabbit baby complement: 2048 and 1024; IgG anti-Hia PS: 3.09 and 0.74 μg/ml; IgM anti-Hia PS: 2.11 and 2.43 μg/ml), we found that both 11–139 and 14–61 isolates were sensitive to killing by the serum in a dose dependent manner, although to a different degree (Fig 2A and 2B). Considering that the amount of rabbit complement used in the serum bactericidal assay constitutes 12.5% of the total reaction volume [14], in the subsequent experiments with endogenous complement, we used the 12.5% serum concentration. No correlation was found between serum bactericidal activity using exogenous versus endogenous sources of complement (Pearson r = 0.14, P = 0.67), or between CH50 Eq U/ml concentration and endogenous bactericidal activity (Pearson r = 0.38, P = 0.27, S1 Table, S1 File).

**Fig 3:**
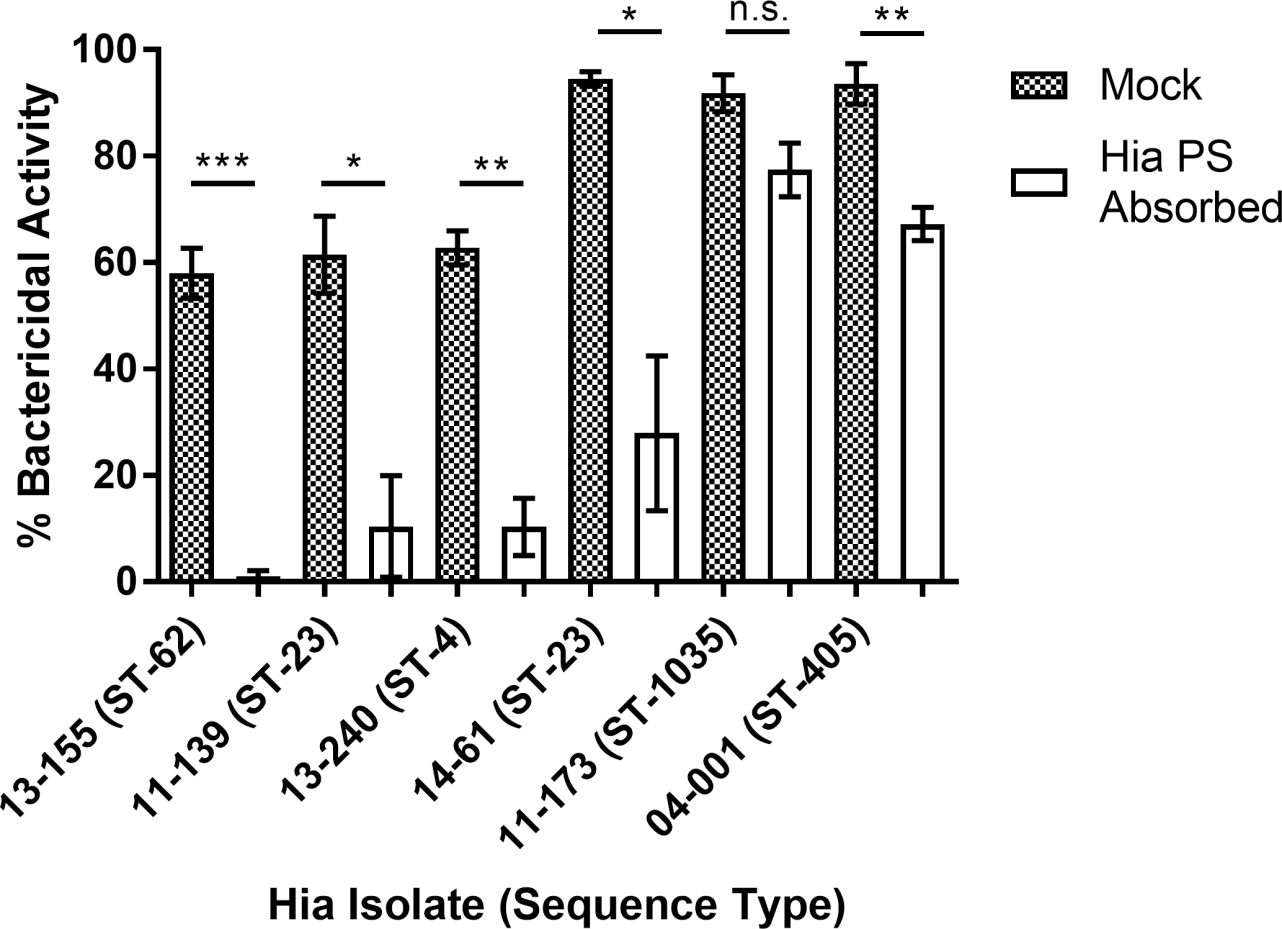
Hia serum sensitivity following Hia polysaccharide (PS) absorption. Absorption of anti-Hia PS antibodies from pooled serum reduced serum sensitivity of Hia isolates. Isolates were incubated with mock-absorbed or Hia PS absorbed sera (12.5%) for 1 hour at 37^o^ C and then viable bacteria were enumerated. Bars indicate mean percent bacterial killing determined by comparing viable bacteria following serum treatment versus heat inactivated serum ±SEM. Statistical significance was determined using Student's two tailed t-test. *P<0.05; ** P<0.01; ***P<0.001; (n.s.), not significant. The graph is representative of three independent experiments, each performed in triplicate, which gave similar results.

As shown on Fig 3, the sensitivity to serum bactericidal effect varied among Hia clinical isolates belonging to different sequence types. However, the pooled serum from First Nations residing in Northwestern Ontario exhibited bactericidal activity towards invasive isolates from Manitoba even if they were genetically unrelated to ST-23 and have not been reported circulating in Northwestern Ontario, such as those belonging to ST-62, ST-4, ST-1035, or ST-405 (the genetic background of various STs is explained in Table 2).

### Hia capsular polysaccharide-specific antibody does not entirely account for the overall antibody-mediated serum bactericidal activity against Hia

As capsular polysaccharide is the major antigenic component of encapsulated bacteria we assessed the contribution of anti-capsular antibody to the serum bactericidal activity via absorbing the pooled serum with purified Hia capsular polysaccharide. Although the absorption did not exhaust the anti-capsular antibody completely, it resulted in a decrease in concentrations of anti-Hia PS specific IgG from 1.92 to 0.49 μg/ml, and specific IgM from 2.27 to 0.54 μg/ml as determined by ELISA.

**Fig 4:**
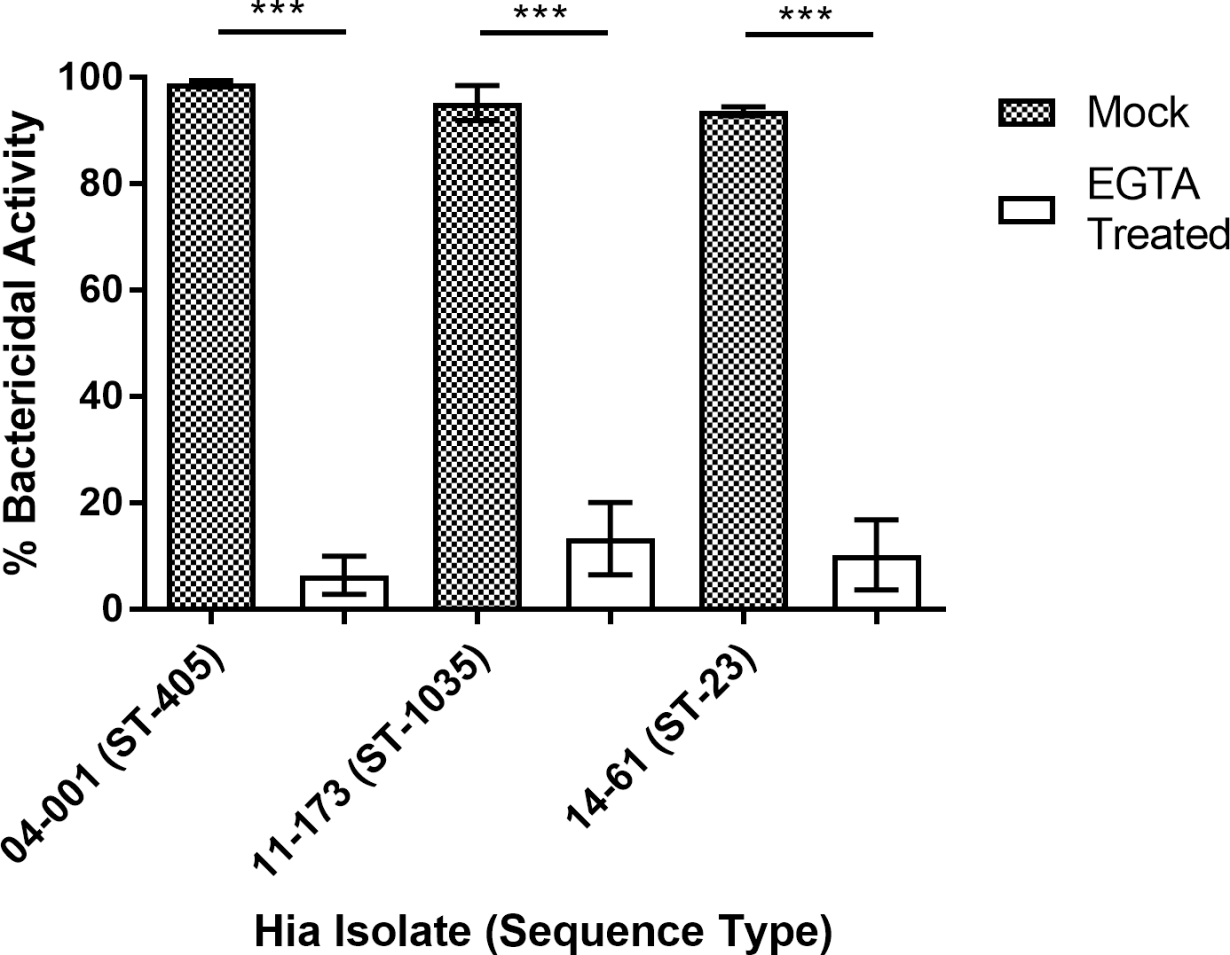
Serum sensitivity of Hia isolates following nullification of the classical complement pathway: The majority of the serum bactericidal activity is antibody mediated (classical complement pathway). Pooled human serum was mock or Mg^2+^EGTA treated and evaluated for bactericidal activity against select Hia isolates. Bars indicate mean percent bactericidal activity compared to heat inactivated sera ±SEM. Statistical significance was determined using Student's two tailed t-test. *P<0.05, ** P<0.01, ***P<0.001. The graph is representative of two independent experiments, each performed in triplicate, which gave similar results.

Polysaccharide-absorbed serum exhibited significantly reduced bactericidal activity (compared to the mock absorbed serum) towards all tested Hia isolates except for 11–173. In the case of Hia 11–173, percent of bacterial killing was insignificantly reduced from 91.86±3.47% to 77.42±5.04% (Fig 3). In addition, the removal of anti-capsular antibody did not abolish the majority of serum bactericidal activity against isolate 04–001 although significantly diminished it from 93.60±3.80% to 67.25±3.16% (Fig 3). Although both invasive Hia isolates 11–173 and 04–001 showed serum sensitivity similar to the non-invasive isolate 14–61, in the case of 14–61, absorption of anti-capsular antibody reduced the serum killing effect over 3 times (from 94.50±1.35% to 27.93±14.54%). As blocking the classical complement activation pathway by treating the serum with Mg^2+^ EGTA drastically reduced its bactericidal activity towards isolates (11–173, 04–001, and 14–61) it was obvious that the effect was antibody-mediated (Fig 4).

### Antibodies specific to Hia lipooligosaccharide are a significant source of serum bactericidal activity against isolates 11–173 and 04–001

As both 11–173 and 04–001 Hia isolates were highly sensitive to antibody-mediated bactericidal effect, which was only partially associated with anti-capsular PS, we further addressed the specificity of antibody accounting for serum bactericidal activity using absorption with other antigenic compounds of *H*. *influenzae*.

**Fig 5:**
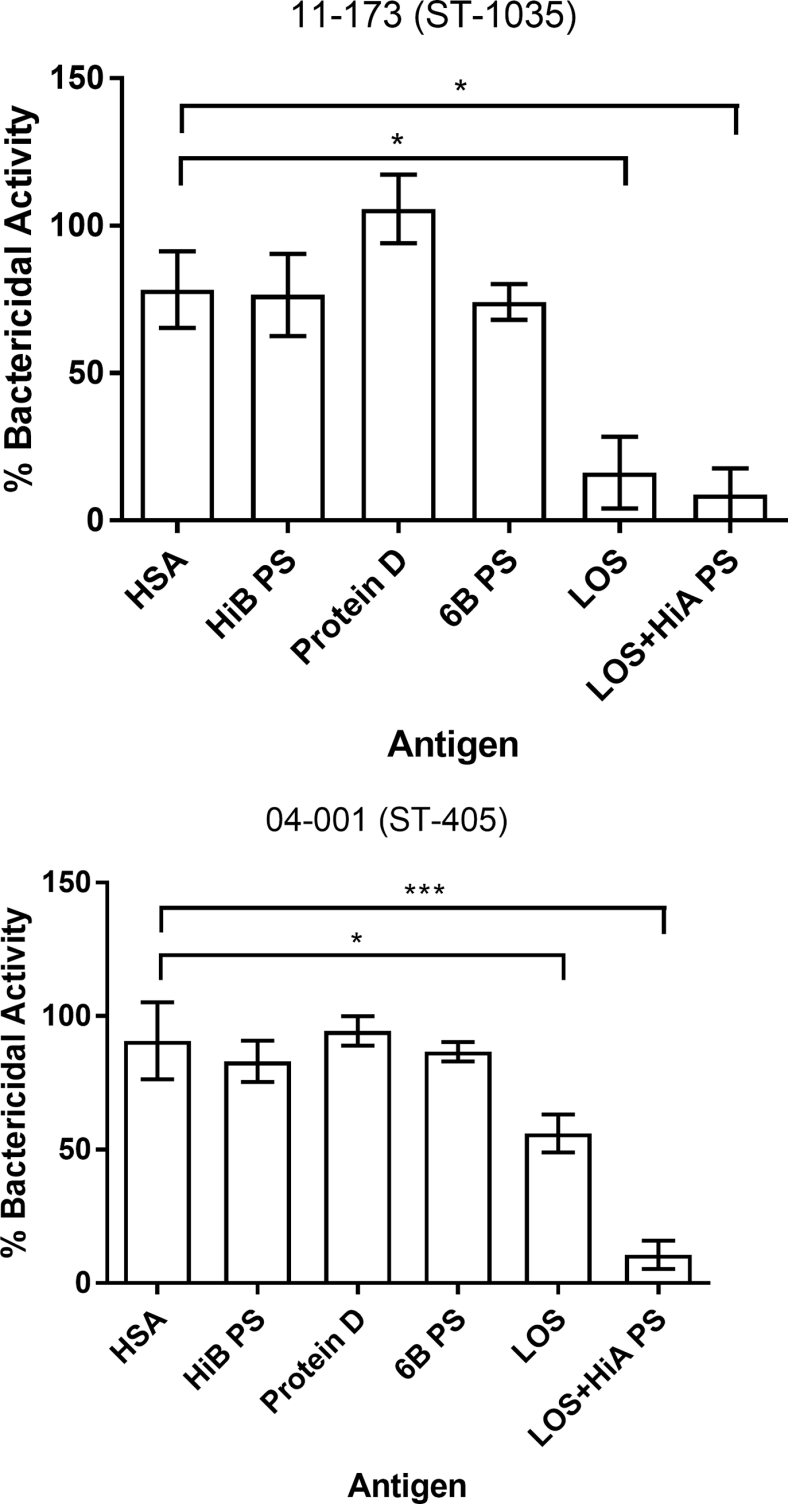
Bactericidal activity of serum following absorption with different antigens: Anti-Hia LOS antibodies are a significant source of serum bactericidal activity against some Hia isolates. The isolates 04–001 (A) and 11–173 (B) were incubated with mock or antigen absorbed pooled human serum. Human serum albumin was used as an irrelevant antigen. Bars indicate mean percent bacterial activity relative to mock absorbed sera ±SEM. Statistical significance was determined using one-way ANOVA with Dunnett's multiple comparison post hoc test. *P<0.05, ** P<0.01, ***P<0.001, HSA = human serum albumin, HiB PS = H. influenzae type b polysaccharide, 6B PS = Pneumococcal polysaccharide 6B, LOS = Hia lipooligosaccharide, LOS+Hia PS = LOS + Hia polysaccharide. The graphs are representative of three independent experiments, each performed in triplicate, which gave similar results.

Serum absorption with capsular PS of either antigenically distinct Hib, or *Streptococcus pneumoniae* serotype 6B, which was previously shown to cross-react with Hia PS [26], did not have any noticeable effect on serum bactericidal activity towards 11–173 or 04–001 Hia isolates (Fig 5A and 5B). Likewise, absorption with protein D, a conserved outer membrane protein of *H*. *influenzae* [27], did not decrease serum bactericidal activity (Fig 5A and 5B). However, absorption of serum with Hia lipooligosaccharide (LOS) almost completely abolished bactericidal activity against Hia 11–173 (Fig 5A) and significantly decreased bactericidal activity against Hia 04–001, which was drastically decreased when Hia LOS and PS were combined (Fig 5B).

These data indicate that antibody specific to LOS can be an important source of serum bactericidal activity against certain Hia clinical isolates.

Interestingly, intravenous IgG (IVIG) prepared from a pool of over a thousand Canadian donors did not exhibit any measurable bactericidal activity against Hia (08–191) when tested with rabbit baby complement (SBA titre 8), and the concentration of anti-Hia PS specific IgG was below the lower detection level of the assay (0.05 μg/ml).

## Discussion

It remains unexplained why certain populations of North American Indigenous people have an increased incidence of invasive Hia disease compared to the general population of Canada and the U.S. [8, 28]. In Canada, three distinct groups of Indigenous peoples are recognized, i.e. First Nations, Inuit and Métis, which have unique histories, languages, cultural practices and spiritual beliefs. The highest incidence rates of invasive Hia disease are currently reported from two regions of Northern Canada, Nunavut and Nunavik, which are populated by Inuit, where the disease mostly affects young children. In 2000–2012, the annual incidence rate of invasive Hia disease in Nunavut was 274.8/100,000 children <1 year, and 61.2/100,000 population of 1–4 year old [29]. The incidence rates for the same age groups during an outbreak of invasive Hia disease in Nunavik in 2010–2013 reached 330.1/100,000 and 191.4/100,000, correspondingly [30].

Northwestern Ontario is a large (526,371.87 km^2^) area of Canada, with population of 231,691 [31]; it has two major urban centres: the City of Thunder Bay and Kenora. Other parts of Northwestern Ontario are largely rural with populations of less than 10,000; 23% of the population are Indigenous (First Nations and Métis). During 2002–2008, in this region, annual incidence rates of invasive Hia disease were in the range of 7.7 to 23.2 per 100,000 children <5 years old, with Hia being predominant among invasive *H*. *influenzae* isolates [12]. In comparison, in the whole province of Ontario, during 2004–2013, Hia represented 5% of all invasive *H*. *influenzae* isolates, and annual incidence rates of invasive Hia disease were between 0.02 and 0.08/100,000 population [32].

In our previous study, we found that both healthy and immunocompromised Indigenous (predominantly First Nations) adults exhibited significantly higher bactericidal antibody titers against Hia than did non-Indigenous adults living in the same geographic area of Northwestern Ontario [14]. These findings suggest that high Hia circulation rates in First Nations communities stimulate the development of antibodies because of an increased exposure of the population to Hia antigens. Indeed, our epidemiological studies have demonstrated that in this region, most cases of invasive Hia disease occur in First Nations [12, 13, 22, 24, 33]. Moreover, our ongoing research in First Nations communities of Northwestern Ontario identified Hia as a common cause of pediatric otitis media and found that approximately 8% of 3–5 year old children carry Hia in the nasopharynx (unpublished observations). Hence, increased circulation rates of Hia may at least partly explain the occurrence of higher bactericidal antibodies in First Nations communities that is in agreement with our present findings of higher titres of bactericidal antibodies in residents of the NWFN compared to non-First Nations residents of Kenora, with both populations living in the same region. As we extended the study to other communities we confirmed our previous findings that generally healthy First Nations adults have higher naturally acquired immunity against Hia than their non-First Nations counterpart. These observations are concordant with the epidemiological data as most cases of invasive Hia disease in this region occur either in young children or immunocompromised individuals who apparently lack protective immunity, but not in healthy adults [12, 13, 22–24].

However, an increased exposure to Hia can hardly explain that the highest bactericidal antibodies to Hia were found in a First Nation (SFN) community with no reported cases of invasive Hia disease although no data on Hia carriage in this population are available. While it may be of importance that this community resides in a geographically different area (Southern Ontario) it is uncertain whether climate or geography may influence the natural immunity. One possibility is that this population is exposed to some cross-reactive environmental antigens which stimulate the production of Hia antibodies. By analogy, cross-reactivity between the capsular polysaccharides of Hib and *Escherichia coli* K100 has been demonstrated by earlier studies [34]. Indeed, the cross-reactivity between capsular polysaccharide antigens of Hia and *Streptococcus pneumoniae* 6B has been described [26]. However, when we tested the possibility that serum bactericidal activity against Hia may be attributed to cross-reactivity with *S*. *pneumoniae* 6B using absorption with 6B capsular polysaccharide antigen, the results were negative.

The origin of bactericidal antibodies against Hia remains obscure. They may be part of the natural antibody repertoire constitutively present in human and other species including newborns and germ-free animals, i.e. without apparent antigenic stimulation [35]. Natural antibodies are predominantly IgM, produced by a subset of B cells (B-1) characterized by the usage of germline-encoded variable immunoglobulin genes (V_H_ and V_L_) without N-region insertions [36]. In our study, we found a greater prevalence of IgM *vs* IgG among anti-Hia PS antibodies, and IgM typically predominates the natural antibody repertoire. Moreover, the Hia antibodies in our study are presumably protective, as they were present in healthy adults who do not typically develop invasive Hia disease while immunocompromised adults have decreased bactericidal antibodies against Hia [14]. A critical role of natural IgM antibodies in the host defense against systemic bacterial infection was demonstrated by earlier studies [37, 38]. Because of its pentameric structure with a central protruding region, IgM has 1,000-fold greater ability to bind C1q, which initiates the classical complement activation, compared to IgG, and hence IgM has a superior capacity to contribute to the bactericidal effect [15]. However, total serum IgG harbours multiple specificities, including antibody directed against non-capsular *H*. *influenzae* antigens that may contribute to serum bactericidal activity. We did not find any correlation between SBA titres and anti-capsular polysaccharide IgG or IgM antibody concentrations (data not shown) that is in line with our previous observations [14]. In contrast to vaccine-induced antibody, in the natural antibody repertoire, antibodies of other specificities in addition to the capsular antigens can contribute to the bactericidal activity, and a lack of correlation between anti-Hia PS capsular antibody concentrations and SBA titres was previously demonstrated [39]. Although in addition to complement activation, serum antibodies have other functions in host defenses against bacterial pathogens, such as opsonisation of bacteria for phagocytosis or agglutination of bacteria, such functions have not been documented for Hia.

If reactivity against Hia is attributed to natural antibodies, the obvious question is why both the serum bactericidal activity and specific IgM concentrations are higher in First Nations than in non-First Nations? Interestingly, a significantly increased antibody response against Hib PS was observed in Canadian Indigenous infants compared to non-Indigenous infants immunized with Hib conjugate vaccine, while no difference in antibody response was observed against the protein vaccine antigen of hepatitis B virus [40]. Although the striking differences in natural immunity against Hia observed in our study between First Nations and non-First Nations adults are not easy to explain, some ethnic dependent peculiarities in the antibody repertoire specific to bacterial polysaccharide antigens may be involved. Keeping this in mind, it will be important to address the potential role of epigenetic factors associated with the social determinants of health, consequences of poor nutrition, and oppression imposed by a history of colonization and residential schools endured by several generations of First Nations in contrast to non-First Nations residents of Canada [41, 42]. Recent studies found that prenatal maternal stress resulted in profound epigenetic changes in the offspring leading to significant psychological and biological outcomes [43, 44]. Interestingly, we found no apparent reactivity against Hia in an IVIG preparation composed of pooled plasma of over 1,000 Canadian donors that may reflect that 1) mainly non-Indigenous donors contributed to this pool and 2) no IgM is present in IVIG. Although the lowest serum bactericidal activity and specific IgM concentrations among all the groups were found in NFNC, interpretation of these data is complicated by the fact that both First Nations groups were considerably younger, and age might be a factor contributing to a decline in natural immunity; this is suggested by a slight, but significant negative correlation between SBA titres, specific IgM concentrations, and age.

While the origin of naturally acquired bactericidal antibodies against Hia is mysterious we made some attempts to characterize them. We found that serum bactericidal activity in First Nations adults extended beyond recognition of Hia strains currently circulating in the region, i.e. clonal type ST-23 [13, 22, 23]. Some isolates we tested have not been identified in the region since observations started in 2002, e.g. strains belonging to ST-62, ST-4, ST-1035, and ST-405. The ST-4 and ST-62 belong to genetically distinct clonal complexes, unrelated to each other and different from members of the ST-23 clonal complex, i.e. ST-23, ST-1035 and ST-405 [19, 45]; nevertheless the sera exhibited bactericidal activity against all these isolates. Importantly, a representative of the ST-4 containing the IS*1016-bexA* partial deletion, which has been associated with enhanced virulence (isolate 13–240), showed sensitivity to the serum bactericidal effect comparable to the isolate 11–139 (ST-23), which circulates in the area [19].

However, the sensitivity of individual isolates to the bactericidal effect considerably varied, potentially reflecting variability in the amount of capsular material in different isolates as differences in capsule thickness may result in different degrees of LOS exposure to antibodies. Indeed, the bactericidal activity against the isolates with relatively higher resistance to serum (13–155, 11–139, and 13–240) was almost entirely attributed to anti-capsular antibody, while the bactericidal activity against relatively more serum-sensitive isolates 11–173 and 04–001 involved non-capsular antibody as demonstrated by the absorption experiments (Figs 3 and 5). Previous studies found that variability in the amount of capsular material expressed by Hib strains determined their capacities to colonize the nasopharynx or invade human cells, and this may be true for Hia [46]. Interestingly, the sensitivity to bactericidal effect differed between invasive and non-invasive isolates of the same ST-23, suggesting that certain virulence factors make some Hia strains more prone to cause invasive disease, even if they share the same molecular-genetic characteristics as assessed by the multilocus sequence typing. Nevertheless, our findings suggest a conserved nature of protective epitopes recognized by bactericidal antibodies that may be explained by the uniform composition of Hia capsular PS, a polymer of glucose and ribitol connected by phosphodiester linkages [47]. Among others, we tested an isolate (11–139, ST-23), of which a polysaccharide antigen was used for the development of Hia vaccine candidate, and this isolate showed similar sensitivity to serum bactericidal effect as 13–155 (ST-62) and 13–240 (ST-4) suggesting that antibodies stimulated by immunization with the antigen from a vaccine candidate strain will be protective against various Hia bacteria [48].

Although capsular polysaccharides represent the major antigenic components of encapsulated bacteria we found that LOS substantially contributes to the natural bactericidal activity against Hia. These findings corroborate our previous data that naturally acquired antibodies specific to LOS are bactericidal against Hib (Eagan) [49]. The contribution of non-capsular antigens to protection against other encapsulated bacteria, i.e. *S*. *pneumoniae* was emphasized by others [50, 51]. These observations suggest that antibodies to non-capsular antigens are an important part of the naturally acquired antibody repertoire against various encapsulated bacteria including Hia. Indeed, our data imply that the bactericidal activity for isolates 11–173 and 04–001 is more dependent on LOS antibodies because of efficient killing with Hia PS absorbed serum (Figs 3 and 5). It is uncertain why the role of LOS-specific antibody in bactericidal activity against Hia depends on the particular Hia isolate and this question deserves further study.

### Limitations

The number of participants in this study were insufficient to complete analysis by age stratified groups. The lack of pediatric data does not allow to the current study to address the onset and development of natural immunity. As we addressed characteristics of natural antibody against Hia in only two First Nation communities, it is impossible to generalize our findings towards other Indigenous populations that have an increased burden of invasive Hia disease, such as different First Nations communities or Inuit Peoples. Although serum bactericidal activity is considered potentially protective against invasive Hia infection, the minimal protective titer has not been determined in animals or humans.

## Conclusions

The results of this research extend and support our previous findings that healthy Canadian Indigenous adults (First Nations) have greater natural immunity against Hia compared to their non-Indigenous counterparts. The origin of natural bactericidal antibodies against Hia remains elusive due to the absence of knowledge regarding the natural history of exposure to Hia across the lifespan. These antibodies may represent part of the overall natural antibody repertoire, which is potentially formed in the population of First Nations under the influence of certain epigenetic factors. The serum bactericidal activity extends to Hia isolates of various clonal groups, which are different from the one circulating in the region, and is mediated by antibodies specific to Hia capsular polysaccharide as well as LOS of both IgM and IgG isotypes. Although the nature of these antibodies deserves further study to understand their origin, the current data suggest that they may represent an important protective mechanism against infections caused by Hia. It will be important to extend the analysis to other Indigenous populations, which have an increased burden of invasive Hia disease, as well as to study natural immunity against Hia in children of various ages.

## Supporting information

S1 Fig Correlation of SBA titres with age for the groups of NWFN, SFN, and NFNK combined. A weak negative correlation of SBA titres with age was detected, i.e. Pearson coefficient of correlation r = -0.2077 (95% CI -0.3645–0.03947; P = 0.016; R2 = 0.04314) for the groups of NWFN, SFN, and NFNK combined.
(TIF)

S2 Fig Correlation of SBA titres with age for the groups of NWFN and SFN combined. A weak negative correlation of SBA titres with age was detected, i.e. Pearson coefficient of correlation r = -0.219 (95% CI -0.3918–0.0313; P = 0.0228; R2 = 0.04797) for NWFN and SFN combined.
(TIF)

S3 Fig Correlation of Hia-specific IgM concentrations with age. A weak negative correlation of Hia-specific IgM concentrations with age in GFN and SFN combined was detected, i.e. Pearson coefficient of correlation r = -0.2947 (95% CI -0.4749–0.09087; P = 0.0053; R2 = 0.08688).
(TIF)

S1 Table Serum bactericidal activity, antibody concentrations, and complement activity of individual sera. ^1^ and ^2^: samples that make up Pool #1 and #2, respectively; Hia, H. influenzae type a; CP, capsular polysaccharide; N/A: sample not analyzed.
(DOCX)

S1 File Raw data of all experiments. (XLSX)
