# Supplementary figures and images for: Characterization of natural bactericidal antibody against Haemophilus influenzae type a in Canadian First Nations: A Canadian Immunization Research Network (CIRN) Clinical Trials Network (CTN) study
Source: PLoS One. 2018 Aug 15;13(8):e0201282. doi: 10.1371/journal.pone.0201282 (PMC6093645; doi:10.1371/journal.pone.0201282)

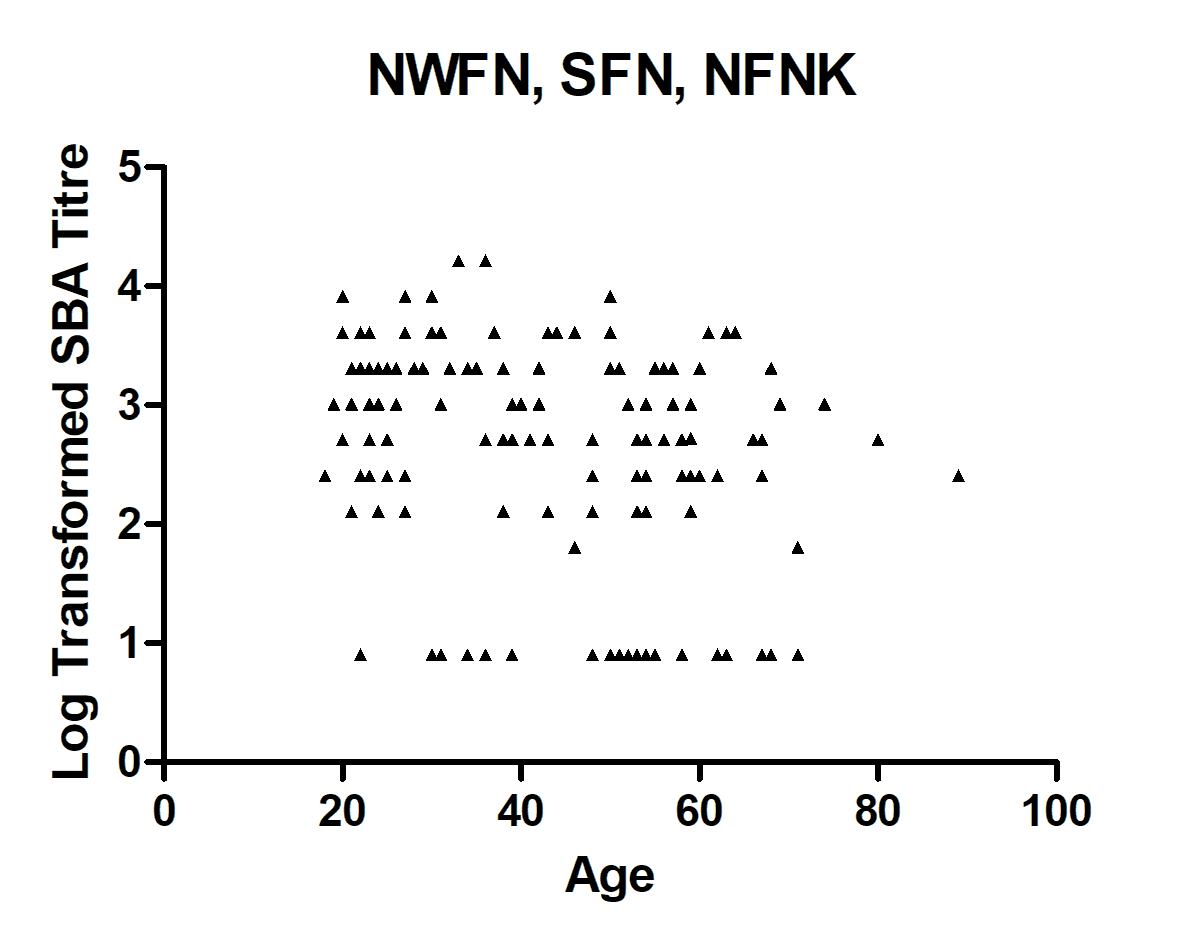

Supplement: S1 Fig — A weak negative correlation of SBA titres with age was detected, i.e. Pearson coefficient of correlation r = -0.2077 (95% CI -0.3645–0.03947; P = 0.016; R2 = 0.04314) for the groups of NWFN, SFN, and NFNK combined. (TIF) [file pone.0201282.s001.tif]

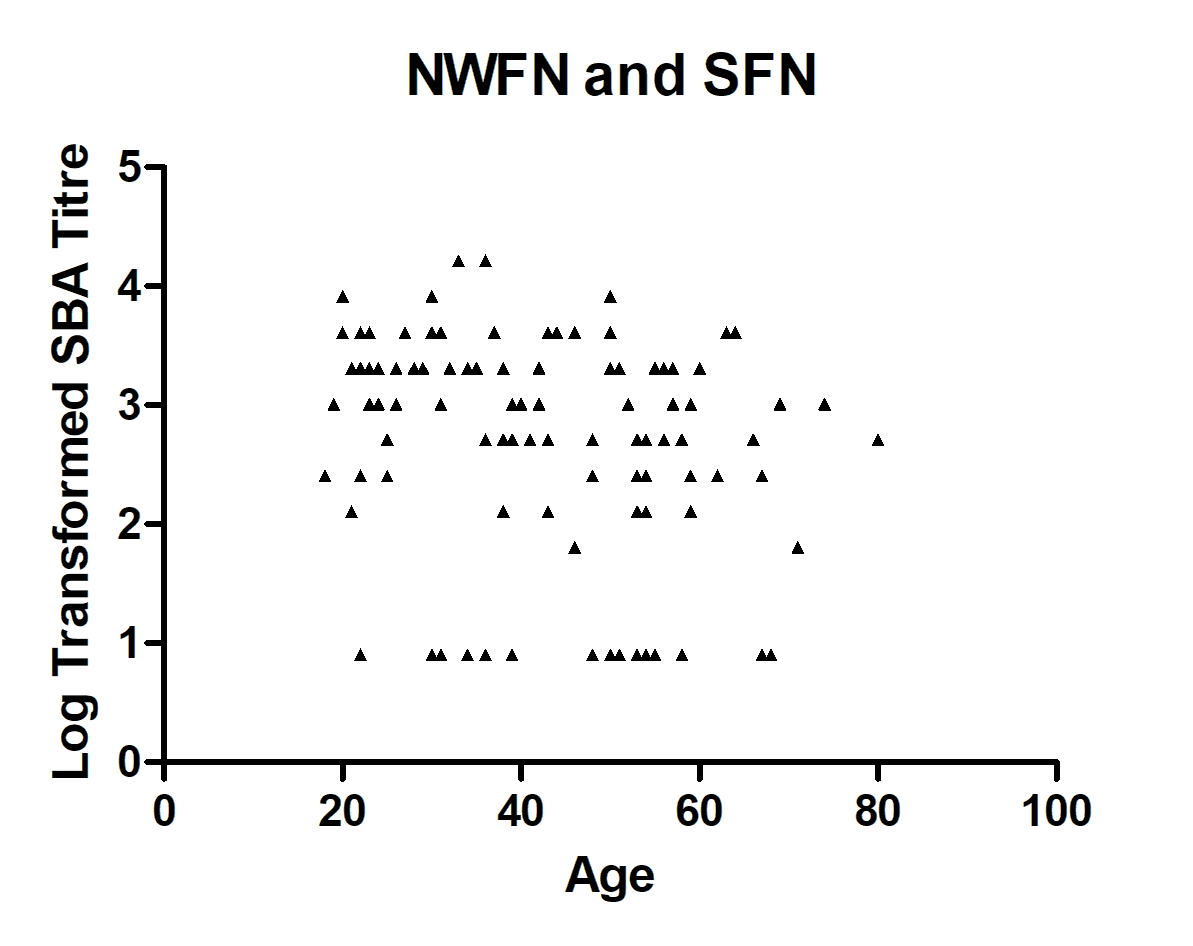

Supplement: S2 Fig — A weak negative correlation of SBA titres with age was detected, i.e. Pearson coefficient of correlation r = -0.219 (95% CI -0.3918–0.0313; P = 0.0228; R2 = 0.04797) for NWFN and SFN combined. (TIF) [file pone.0201282.s002.tif]

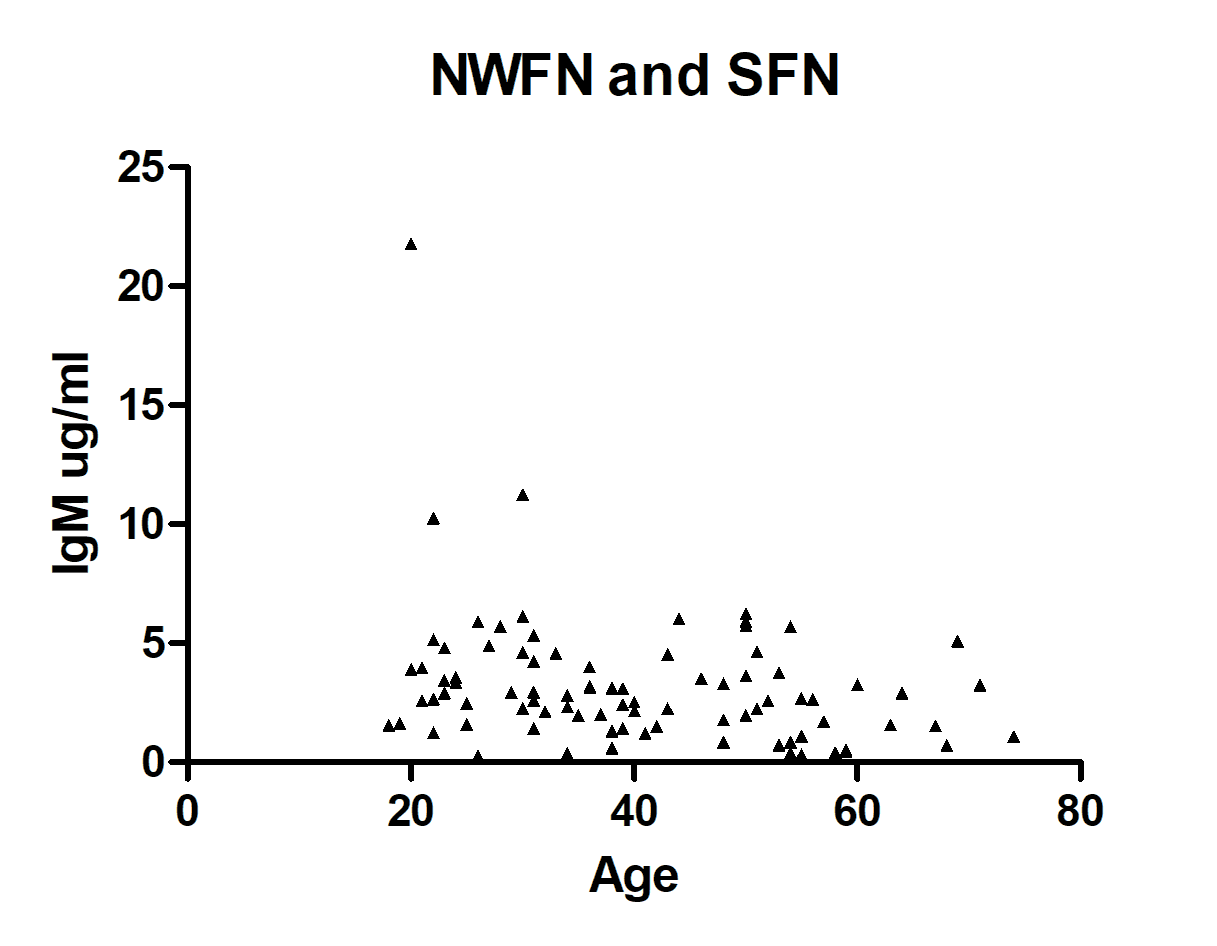

Supplement: S3 Fig — A weak negative correlation of Hia-specific IgM concentrations with age in GFN and SFN combined was detected, i.e. Pearson coefficient of correlation r = -0.2947 (95% CI -0.4749–0.09087; P = 0.0053; R2 = 0.08688). (TIF) [file pone.0201282.s003.tif]
