# Supplementary material for: Characterization of natural bactericidal antibody against Haemophilus influenzae type a in Canadian First Nations: A Canadian Immunization Research Network (CIRN) Clinical Trials Network (CTN) study
Source: PLoS One. 2018 Aug 15;13(8):e0201282. doi: 10.1371/journal.pone.0201282 (PMC6093645; doi:10.1371/journal.pone.0201282)
Supplement: S1 Table — 1 and 2: samples that make up Pool #1 and #2, respectively; Hia, H. influenzae type a; CP, capsular polysaccharide; N/A: sample not analyzed. (DOCX) [file pone.0201282.s004.docx]

S1 Table. Serum bactericidal activity, antibody concentrations, and complement activity of individual sera

| Sample ID | Age | Sex | SBA titre (Exogenous complement) | Anti-Hia CP IgG (μg/ml) | Anti-Hia CP IgM (μg/ml) | Bactericidal activity (Endogenous complement)  (%) | Complement activity  (CH50 U Eq/ml) |
| --- | --- | --- | --- | --- | --- | --- | --- |
| w3 | 23 | F | 1024 | 1.03 | 8.72 | 92.84 | 133.03 |
| w7^2^ | 56 | M | 8 | 6.74 | 0.50 | 94.09 | 71.05 |
| w9 | 50 | F | 8 | 3.31 | 1.97 | 56.41 | 72.50 |
| w14 | 54 | M | 1024 | 11.61 | 0.44 | 96.95 | 94.87 |
| w15 | 49 | M | 2048 | 0.50 | 0.72 | 96.41 | 78.16 |
| w16^1^ | 60 | F | 2048 | 3.09 | 2.11 | N/A | 106.18 |
| w17^1^ | 42 | F | 1024 | 0.74 | 2.43 | N/A | N/A |
| w20^2^ | 45 | F | 2048 | 1.21 | 5.78 | 100 | 73.03 |
| w24^2^ | 53 | F | 1024 | 0.55 | 3.72 | 98.55 | 141.32 |
| w29 | 60 | F | 1024 | 0.12 | 2.75 | 89.42 | 100.53 |
| w33 | 31 | M | 4096 | 1.86 | 2.54 | 94.42 | 100.39 |
| g7^2^ | 40 | F | 1024 | 1.19 | 2.15 | 100 | 155.26 |
| Descriptive statistics | Mean: 46.92  Median: 49.5  Range: 23-60 | Ratio M:F  0.33 | Geometrical Mean: 608.9  95% Confidence Intervals: 163.3,2271 | Geometrical Mean: 1.39  95% Confidence Intervals: 0.63,3.07 | Geometrical Mean: 2.02  95% Confidence Intervals: 1.13,3.60 | Geometrical Mean: 90.85  95% Confidence  Intervals: 80.39,102.7 | Geometrical Mean: 98.77  95% Confidence Intervals: 81.88,119.1 |

^1^ and ^2^: samples that make up Pool #1 and #2, respectively

Hia, H. influenzae type a; CP, capsular polysaccharide

N/A: sample not analyzed
